# Effect of salt solution wash versus warm water wash for leucorrhea symptoms among postmenopausal women

**DOI:** 10.6026/973206300213730

**Published:** 2025-10-31

**Authors:** Siva Subramanian N, Sonalben Sendhabhai Nadiya, Sandipkumar Rajeshbhai Patel, Gnanadesigan Ekambaram, Venkateshwaran T, Anuradha M, Dhana Priya G, Rama lakshmi G, Mahalakshmi B

**Affiliations:** 1 Department of Psychiatric Nursing, Nootan College of Nursing, Sankalchand Patel University, Visnagar, Gujarat - 384315, India; 2 Department of Obstetric & Gynacolagil Nursing, Nootan College of Nursing, Sankalchand Patel University, Visnagar, Gujarat - 384315, India; 3 Department of Biochemistry, Dr.N.D Desai Faculty of Medical Science and Research, Dharamsinh Desai University, Nadiad, Gujarat, India; 4 Department of Physiology, Nootan Medical College and Research Centre, Sankalchand Patel University, Visnagar, Gujarat, India; 5 Department of Psychiatric Nursing, PPG college of Nursing, Coimbatore, The Tamilnadu DR. M.G.R Medical University, Chennai, India; 6 Department of Community Health Nursing, KMCH College of Nursing, Coimbatore, The Tamilnadu DR. M.G.R Medical University, Chennai, India; 7 Department of Medical surgical Nursing, Madurai Apollo college of Nursing, Madurai, The Tamilnadu DR. M.G.R Medical University, Chennai, India; 8 Department of community health Nursing, SGRRIM & HS College of Nursing, Shri Gururam Rai University, Dehradun, Uttarakhand - 248001, India; 9 Department of Pediatric Nursing, Nootan College of Nursing, Sankalchand Patel University, Visnagar, Gujarat - 384315, India

**Keywords:** Leucorrhea, salt solution wash, warm water wash, postmenopausal women, vaginal hygiene, symptom reduction

## Abstract

Leucorrhea is a common gynecological concern among postmenopausal women, often resulting from infections, hormonal changes, or hygiene issues. Therefore, it is of interest to compare the effectiveness of salt solution wash versus warm water wash in reducing leucorrhea symptoms. Hence, sixty postmenopausal women were divided equally into two groups for a 7-day intervention. Results showed a significant reduction in symptoms in the salt wash group (p < 0.05), while warm water showed minimal improvement. Demographic factors such as residence, diet and hygiene knowledge were associated with outcomes. Salt wash demonstrated superior antimicrobial and cleansing effects. It offers a cost-effective, accessible hygiene intervention in resource-limited settings.

## Background:

Leucorrhea, characterized by excessive or abnormal vaginal discharge, is a prevalent concern among women, particularly in the postmenopausal stage. It often results from hormonal imbalance, genital infections, or poor perineal hygiene, leading to itching, foul odor and social discomfort [1]. Traditional hygiene interventions such as warm water washes are commonly employed due to their simplicity and accessibility. Warm water soothes irritation, improves local circulation and reduces inflammation in the genital area [2]. Salt solution washes offer a potential advantage because the antibacterial and osmotic properties of salt help reduce microbial load and draw out fluids [3]. A study comparing salt solution with warm water among postmenopausal women reported that the salt group had greater reductions in itching and discharge levels after intervention (Angel, 2014) [4]. Research into mouth rinses after oral surgery found no significant differences in bacterial control between warm saline, salt water and plain water, indicating salt water is at least as effective as other basic antiseptics [5]. Salt-based cleansing methods are used in other fields due to their proven efficacy in reducing microbial contamination [6]. Brine reuse strategies are increasingly applied for sustainable waste management, supporting its use as a low-cost intervention [7]. Salt solution as a local wash is also affordable and culturally acceptable, making it feasible for use in low-resource settings [3]. Therefore, it is of interest to identify a simple, effective and accessible intervention to promote women's reproductive hygiene and well-being.

## Methodology:

This study followed a quantitative, quasi-experimental two-group pre-test and post-test design to compare the effectiveness of salt solution versus warm water wash on leucorrhea symptoms among postmenopausal women in Visnagar City.

## Population and Sampling:

The study targeted postmenopausal women with leucorrhea symptoms. Using purposive sampling, 60 eligible women were selected and equally divided into two experimental groups:

[1] Group I (n=30): Received salt solution wash

[2] Group II (n=30): Received warm water wash

Inclusion criteria included postmenopausal women experiencing leucorrhea, willing to participate. Exclusion criteria ruled out those with chronic conditions or pregnancy.

## Data collection tools and procedure:

Data was collected using a validated structured questionnaire and symptom checklist covering nine leucorrhea symptoms. Scores classified severity as:

[1] 0-3: Mild

[2] 4-6: Moderate

[3] 7-10: Severe

Each group underwent a pre-test, followed by 7 days of intervention (salt solution or warm water wash) and a post-test on the 7th day.

## Data analysis:

[1] Descriptive statistics (mean, SD, percentage) summarized demographics and symptom levels.

[2] Inferential statistics:

1) Paired t-test: Within-group pre- and post-test comparison

2) Chi-square test: Association between symptom levels and demographic variables

## Results:

Table 1 (see PDF) presents the demographic characteristics of the 60 postmenopausal women included in the study equally divided between Group I (Salt Solution Wash) and Group II (Warm Water Wash). The majority of participants in both groups were aged 24-31 years, married, had primary education and lived in urban areas. Most women were non-vegetarian and employed in private jobs. A large portion reported monthly incomes between ₹10,001-20,000. These baseline characteristics were relatively balanced across both groups, supporting comparability for intervention assessment. Table 2 (see PDF) compares the mean symptom scores of leucorrhea before and after intervention in both experimental groups. Group I (Salt Wash) showed a significant reduction in symptoms (t = 13.54, p < 0.05), while Group II (Warm Water) exhibited a slight, non-significant decrease (t = 1.893). This indicates that salt solution wash was more effective in alleviating symptoms. Figure 1 (see PDF) illustrates the changes in leucorrhea severity levels (mild, moderate, severe) before and after intervention in both groups. Group I (Salt Wash) showed a significant improvement, with 90% of participants shifting to mild symptoms post-test. In contrast, Group II (Warm Water Wash) had only a moderate shift, with 60% still experiencing moderate symptoms after the intervention. This visual comparison highlights the greater effectiveness of salt solution wash in reducing symptom severity. Table 3 (see PDF) shows the relationship between post-test leucorrhea symptom levels and demographic variables in both intervention groups. In both Salt Wash and Warm Water Wash, statistically significant associations (p < 0.05) were found with place of residence, dietary pattern, age at menarche and source of knowledge. Other variables such as age, marital status, education, occupation, income and menstrual cycle pattern showed no significant association (p > 0.05) in either group. This indicates that lifestyle and early-life factors may influence treatment outcomes more than current socioeconomic status.

## Discussion:

The present study investigated the comparative effectiveness of salt solution wash and warm water wash in alleviating symptoms of leucorrhea among postmenopausal women. The findings revealed a significant reduction in symptom severity among participants in the salt solution group, with 90% reporting mild symptoms post-test, as opposed to only 40% in the warm water group. This supports the hypothesis that salt solution offers superior relief through antimicrobial, anti-inflammatory and cleansing effects. Additional evidence from biomedical and clinical research reinforces the antimicrobial and therapeutic benefits of salt-based interventions. Saksono (2010) demonstrated that salt washing significantly reduces impurities and enhances stability, a finding that supports the biological efficacy of salt in therapeutic settings [8]. Furthermore, Dikke and Ruzaeva (1993) reported the effective use of mineral salt concentrate "Shirsal" in treating chronic inflammatory gynecological conditions [9]. In a comparative study, Collins *et al.* (2021) found that saltwater rinses exhibited anti-inflammatory effects comparable to chlorhexidine after periodontal surgery, indicating salt's broader utility in mucosal inflammation [10]. Salt has also shown synergy with anti-inflammatory agents. A study by Muñoz *et al.* (2018) found that high salt concentrations significantly enhanced the microbicidal activity of ibuprofen, suggesting salt's role in modulating bacterial membrane potentials to increase drug efficacy [11]. Moreover, Pryme (1974) showed that salt wash treatments stimulated immunoglobulin synthesis *in vitro*, suggesting salt's capacity to enhance immune responses [13]. Further validation comes from studies in biomedical science where salt washes stimulate immune-related processes. For instance, the salt wash of polysomes *in vitro* promoted the synthesis of immunoglobulins, suggesting that salt enhances cellular functional responses [12]. In another example, Amir *et al.* (2022) demonstrated that parameters in salt washing also improve salt quality and sanitation applicability, linking industrial findings to personal hygiene applications [13]. The present study also examined demographic factors and their association with symptom reduction. Place of residence, dietary pattern, age at menarche and the source of hygiene knowledge were significantly associated with treatment outcomes.

Urban residence was linked to better outcomes, likely due to enhanced access to hygiene facilities and education, aligning with klein *et al.* (2024), who found that bacterial vaginosis prevalence differed significantly between urban and rural populations in Kenya [14]. This finding is further supported by recent studies in India. Singh *et al.* (2022) highlighted the high prevalence of RTIs in urban slums, yet stronger health-seeking behavior due to better awareness [15], while Dhakate *et al.* (2025) noted that urban women were more likely to seek treatment for RTIs than rural counterparts despite similar prevalence [16]. Dietary habits also influenced outcomes; non-vegetarian women had higher symptom persistence, echoing Jansåker *et al.* (2022), who linked regional dietary variations to higher infection rates [17]. Hygiene education from healthcare professionals was more effective than informal sources, as supported by Noyes *et al.* (2017), who found a strong link between professional education and improved menstrual hygiene outcomes [18]. Moreover, health education has been shown to significantly improve awareness and preventive practices related to leucorrhea. Kaur and Kapoor (2014) found that structured information improved perceptions in low-literacy communities [19]. Nasution *et al.* (2024) demonstrated the effectiveness of the "Make a Match" educational method in enhancing preventive behavior among adolescents [20]. International research echoes these patterns. Bahram *et al.* (2009) in Iran, Ekpenyong and Davies (2013) in Nigeria and Putri and Kusumawardhani (2021) in Indonesia all found significant links between hygiene practices, education level and prevalence of vaginal infections [21, 22-23]. Similar findings were observed by Khan *et al.* (2017) in India, where the use of cloth and douching practices were strongly associated with bacterial vaginosis [24]. These findings underscore the importance of addressing both treatment and context. While salt wash demonstrated greater clinical efficacy, its effectiveness is also influenced by socioeconomic and educational factors. Public health programs should integrate low-cost interventions like salt washes with structured hygiene education, especially in resource-limited settings.
